# Supplementary material for: Toward patient-centered tuberculosis preventive treatment: preferences for regimens and formulations in Lima, Peru
Source: BMC Public Health. 2021 Jan 11;21:121. doi: 10.1186/s12889-020-10098-5 (PMC7802335; doi:10.1186/s12889-020-10098-5)
Supplement: Supplementary file 1 — Additional file 1. [file 12889_2020_10098_MOESM1_ESM.pdf]

INTERVIEW GUIDE:
FOCUS GROUPS ON PREVENTIVE TREATMENT REGIMEN PREFERENCES

Introduction:
Tuberculosis (TB) is a disease spread by a bacteria found in the air. When the bacteria enter the body, the person can be infected. Some people get very sick quickly with TB disease. But many people stay with the infection for months or years, without getting sick. When this occurs, the bacteria are alive in the body, but are not active. However, when the immune system is weakened by stress or other illness, the bacteria become active and the person can develop TB disease.

For people who have TB infection, it is possible to give treatment to kill the bacteria and not develop TB disease in the future. The purpose of this group conversation is to hear your views on this treatment.

Ask if participants have questions about TB infection and disease, and answer questions.

Vote for regimen choice:
Currently, there are four options recommended by the World Health Organization for the treatment of tuberculosis infection. I will show you the medications and describe each option. Then I would like you to write down on a paper which medicine you would take if you had TB infection. Please do not write your name on the paper.

Hand out ballots. Present table (handout or projected slide), describe duration of treatment, doses, and number of pills per day. Show pills. Collect ballots.

Focus group discussion:

Ask each participant for the reason behind his or her choice. Encourage discussion among participants, but be sure each participant has an opportunity to answer individually.

**Main question**	**Probes**
What are participant's main reasons for regimen preference?	"What were the reasons for your choice?"
	"What options do you think your friends or family members would prefer? Why?"
How do participants feel about duration of treatment?	"How do you feel about the length of time of treatment? 3 months, 4 months, or 6 months?"
How do participants view trade-offs between dosing and pill burden?	If 3HP is chosen: "Does it concern you that this option would make you take more pills at a time?"
	If 3HP is not chosen: "Why did you a regimen that requires pills every day, instead of 3HP, which is only once per week?"
