# Supplementary material for: Toward patient-centered tuberculosis preventive treatment: preferences for regimens and formulations in Lima, Peru
Source: BMC Public Health. 2021 Jan 11;21:121. doi: 10.1186/s12889-020-10098-5 (PMC7802335; doi:10.1186/s12889-020-10098-5)
Supplement: Supplementary file 2 — Additional file 2. [file 12889_2020_10098_MOESM2_ESM.pdf]

**SUPPORTING QUOTES**

Group 1: adults 18–45 years old, asked about preferences for themselves
Group 2: adults 46-70 years old, asked about preferences for themselves
Group 3: caregivers, asked about preferences for their children

## Theme: Memory and medication fatigue are barriers to completing 6H compared to shorter regimens

| Group | Supporting quote |
|---|---|
| 1 | We know that although we may say that we will be responsible for taking [the medications], a person always misses this responsibility at times. At least once, something will always happen by chance. Although it may be six months, a person will forget, even if it is only once; but in six months they may forget ten times, twenty times – yes, at times, thirty days. |
| 1 | Facilitator: What were the reasons for your choice?<br>Participant: Because it a short time, a short time. Because at times, knowing a person, taking six months one gets tired of taking [medications], one forgets. And that's a short time, right? Three months is faster too. |
| 1 | [Referring to a brother-in-law who is taking 6H]<br>At times he gets tired of it and does not want to take it, but he has to continue. Yes, I would like three months and nothing more. |
| 3 | Three months is good…sometimes one gets bored having to do the same thing every day. Just like my daughter – the one who is on treatment – she says to me, "Papa, it's so long." |

## Theme: Difficulty in remembering to take daily medications, weekly dosing would be easier

| Group | Supporting quote |
|---|---|
| 1 | Because if it is daily, there are days when obviously you will forget, and you will miss the treatment that you are carrying. However, once a week, it is more effective and better. |
| 2 | Because me, I would forget every day. But if it is once a week, I will mark it down and just take it. |
| 2 | Participant: It would be easier to take the nine pills weekly.<br>Facilitator: Even though it is more pills?<br>Participant: Yes, it is more pills weekly; it is okay because it would be only one time, and I can forget about taking it again. |
| 2 | Participant: Well, for me, because once, this pill I am taking – there are three of them – I once took twice in the same day. Thank God nothing happened to me.<br>Facilitator: But was it because you forgot, or…<br>Participant: I had forgotten. After lunch, I took the three. And later, again, I took all three again, and they were the same. And now, as I take other pills, there was confusion. Thank God nothing happened to me. |

**Theme: Concern about side effects**

| Group | Supporting quote |
|---|---|
| 1 | Because when my brother-in-law, when he takes [6H], he tells me that it gives him nausea, or maybe [affects] his liver. Well, I would like to take three months and no more. |
| 1 | There are people with liver problems, and taking pills every day will have a greater effect on their fatty liver disease, while taking them once per week would not cause much. |
| 2 | Participant: I don't know, because as I told you, I take other pills. For diabetes, for my heart, and for my head, there are three medications.<br>Facilitator: And with respect to these options that I have mentioned to you, which one would you agree to take?<br>Participant: Whichever does not affect the other medications that I am taking. |
| 3 | [Referring to 3HP]<br>It is many pills for his little stomach, and more than anything I consider the health of the child, the little one, that nothing should harm him and nothing should irritate his stomach. |

**Theme: Preference for avoiding interference with daily life**

| Group | Supporting quote |
|---|---|
| 1 | [Referring to 3HP]<br>Three months compared to six – it is obviously less, right? And it gives you more time to do all your activities, to recuperate. |
| 1 | Everyone has different routines, everyone has different routines for work, whether it be for studies, whether it be for leisure, or whatever they spend their time doing, right? They would choose [3HP] because if it is once per week…it would prevent them from saying 'at such a time I have to go do something.' They would simply say, 'ah, I have to do this today, and tomorrow no' and from then, not until the following week. |

**Theme: Child-friendly formulations are easier for children to take**

| Sub-theme | Group | Supporting quote |
|---|---|---|
| Children have difficulty taking pills | 3 | There are children who cannot take the pill. My son suffers taking it. Although he chews it, the bitter flavor stays in his mouth, and he cries. |
| | 3 | Facilitator: And why would you prefer daily and not, for example, once per week?<br>Participant: Because I know he will not take it. My son will not take it. Or he may suddenly spit it out. He will not swallow.<br>Facilitator: No? But it is once a week?<br>Participant: But he will not swallow the pill. |
| Child-friendly formulations would be easier to take than pills for children | 3 | There are children who cannot swallow pills, and when they chew them, the flavor or the grittiness of the pill stays in their mouth, and they do not swallow. However, in a syrup, he would swallow. It is like taking paracetamol, right? – the syrup that they are used to as children. Well, on the other hand, an older child already understands that he has to take pills. |
| | 3 | Participant: I, as I have three children who are taking pills, of which the last is 5 years old and takes them well, the second also. But the third, who is 10 years old, she chews the pills and does not swallow. However, having fruit flavors, I think yes, she could take them.<br>Facilitator: So for you, it is because of the taste that they would be able to take them then.<br>Participant: Yes, for my daughter; although she chews everything, she does not take [the medication] quickly. |
| | 3 | In my case I also chose [3HR] because I see that it is the one that will make it easy to take. I speak of my son and my nephew, it would be easier for them to take. Because as it is, with [6H], my nephew suffers a lot; in the case of my son, no, because he can take it normally. But my nephew, who is 4 years old, has difficulty, he does not want to take it. And for this reason, I prefer [3HR] because I know that with the flavor of the pill, they can take it normally. |
| | 3 | Partly because of the flavor, and partly because this one is three months – for these reasons I chose [3HR], for the taste more than anything. Because I know that children – most children – will take it. I did not pick [3HP]; [3HP] also seems good, a good option, because it is once a week. But the problem seems to me that in the case of children 5 years old and under, they will not take it. If it were for a 10-year-old, yes… well no, it seems like a lot of pills. But [3HR], yes, it seems to me a good choice for children. [4R], no, because the syrup, as they say, has a bad taste. And [6H], two pills, no; children will not swallow them. For these reasons I have picked [3HR], which is more or less like an effervescent tablet. |
| Children will not take a bad-tasting medicine | 3 | [Referring to rifampin syrup]<br>I have tried a little, and I know that they won't take it either. I have tried it. This is what they have given to my daughter – the one who is on treatment. |
| | 3 | But there are children who do not like fruit flavors, so now if you dilute it in a quantity of water – that is how it would be, right? – for them to finish, they will not. |
